# An approach to lifting self-isolation for health care workers with prolonged shedding of SARS-CoV-2 RNA

**DOI:** 10.1007/s15010-020-01530-4

**Published:** 2020-10-06

**Authors:** H. Laferl, H. Kelani, T. Seitz, B. Holzer, I. Zimpernik, A. Steinrigl, F. Schmoll, C. Wenisch, F. Allerberger

**Affiliations:** 1 4th Medical Department with Infectious Diseases and Tropical Medicine, Kaiser Franz Josef Hospital, 1100 Vienna, Austria; 2 Austrian Agency for Health and Food Safety (AGES), Institute for Veterinary Disease Control Mödling, 2340 Mödling, Austria

**Keywords:** COVID-19, SARS-CV-2, Infectiousness, Health care worker, Isolation

## Abstract

**Purpose:**

According to the European Public Health Authority guidance for ending isolation in the context of COVID-19, a convalescent healthcare worker (HCW) can end their isolation at home and resume work upon clinical improvement and two negative RT-PCR tests from respiratory specimens obtained at 24-h intervals at least 8 days after the onset of symptoms. However, convalescent HCWs may shed SARS-CoV-2 viral RNA for prolonged periods.

**Methods:**

40 healthy HCWs off work because of ongoing positive RT-PCR results in combined nasopharyngeal (NP) and oropharyngeal (OP) swabs following SARS-CoV-2 infection were invited to participate in this study. These HCWs had been in self-isolation because of a PCR-confirmed SARS-CoV-2 infection. NP and OP swabs as well as a blood sample were collected from each participant. RT-PCR and virus isolation was performed with each swab sample and serum neutralization test as well as two different ELISA tests were performed on all serum samples.

**Results:**

No viable virions could be detected in any of 29 nasopharyngeal and 29 oropharyngeal swabs taken from 15 long-time carriers. We found SARSCoV- 2 RNA in 14/29 nasopharyngeal and 10/29 oropharyngeal swabs obtained from screening 15 HCWs with previous COVID-19 up to 55 days after symptom onset. Six (40%) of the 15 initially positive HCWs converted to negative and later reverted to positive again according to their medical records. All but one HCW, a healthy volunteer banned from work, showed the presence of neutralizing antibodies in concomitantly taken blood samples. Late threshold cycle (Ct) values in RT-PCR [mean 37.4; median 37.3; range 30.8–41.7] and the lack of virus growth in cell culture indicate that despite the positive PCR results no infectivity remained.

**Conclusion:**

We recommend lifting isolation if the RT-PCR Ct-value of a naso- or oropharyngeal swab sample is over 30. Positive results obtained from genes targeted with Ct-values > 30 correspond to non-viable/noninfectious particles that are still detected by RT-PCR. In case of Ct-values lower than 30, a blood sample from the patient should be tested for the presence of neutralizing antibodies. If positive, non-infectiousness can also be assumed.

## Introduction

In Austria, coronavirus disease 2019 (COVID-19) causing severe acute respiratory syndrome coronavirus 2 (SARS-CoV-2) was first documented in January 2020 [1]. As the disease progressed all over Austria, increasing numbers of infected health care workers (HCWs) contributed to long periods of absence in the system-relevant infrastructure, placing an unprecedented strain on the healthcare systems. Current guidance (by ECDC [2]) regarding ending isolation for HCWs requires two negative real-time reverse transcriptase-polymerase chain reaction (RT-PCR) tests from respiratory specimens gained at 24 h interval, taken at least 8 days after the onset of symptoms. However, prolonged SARS-CoV-2 RNA shedding with a median duration of 53 days and a maximum of 83 days has been reported recently in 36 patients by Li et al. [3]. In another study, median viral shedding duration of 20 days was observed with a maximum of 37 days [4]. In the context of HCWs, these facts lead to long periods of sick leave, potentially causing staff shortage in hospitals and a potential overload of the healthcare service. As there is no consistent evidence that viral nucleic acid detection of SARS-CoV-2 via RT-PCR automatically equates to infectivity, these periods of absence may be longer than necessary. The aim of our evaluation was to determine if the infectious virus can be isolated from upper respiratory tract samples from clinically recovered HCWs with continued SARS-CoV-2 RNA shedding and to match these data with the presence of SARS-CoV-2 neutralizing antibodies in serum.

## Methods

### Study design and participants

In Austria, COVID-19 has been a reportable disease since 27 January 2020. Between April 30 and June 11, 2020, 40 healthy HCWs off work because of ongoing positive RT-PCR results in combined nasopharyngeal (NP) and oropharyngeal (OP) swabs following SARS-CoV-2 infection were invited by the Viennese public health authority to participate in this study. These HCWs had been in self-isolation because of a PCR-confirmed SARS-CoV-2 infection. Persons with positive RT-PCR result were invited for follow-up visits, of which the last one took place on 29 June 2020. At each appointment, NP and OP swabs as well as a blood sample were collected from each participant. RT-PCR and virus isolation was performed with each swab sample and serum neutralization test as well as two different ELISA tests were performed on all serum samples.

This study was approved by the local research ethics committee (approval number EK 20–131-VK).

## Material collection and processing

### SARS-CoV-2 RNA detection by RT-PCR

RNA was extracted from 200 µl of NP and OP swab supernatants using a commercial kit (BioExtract® SuperBall®, BioSellal, France) and the KingFisher™ Flex Purification System (Thermo Fisher Scientific, USA). Negative extraction controls (nuclease-free water) were prepared alongside clinical samples to monitor for potential cross-contamination. Detection of SARS-CoV-2 RNA was performed using a commercial primer/probe mix (LightMix® Modular SARS and Wuhan CoV E-gene; TIB Molbiol, Germany) and SuperScript™ III Platinum® One-Step Quantitative RT-PCR System with ROX (Thermo Fisher Scientific, USA) on the ABI7500Fast system (Thermo Fisher Scientific, USA). Assays were performed as duplex real-time RT-PCR reactions, also targeting β-actin mRNA as extraction control (Toussaint et al. 2007 [5]). Nuclease-free water and a synthetic RNA control provided with the primer/probe mix were included as respective no-template-control (NTC) and positive control (PC). A SARS-CoV-2 Ct-value of > 42 was considered a negative result.

### Cell lines and viruses

Vero 76 clone E6 cells (CCLV-RIE929, Friedrich-Loeffler-Institute, Riems, Germany) were cultured in a minimum essential medium Eagle (E-MEM) with Hank's balanced salt solution (HBSS) (BioWhittaker, Lonza, Szabo Scandic, Austria), supplemented with 10% fetal bovine serum (Corning, Szabo Scandic, Austria) (FBS) and were used to titrate virus preparations and neutralization assays. Fifty-percent tissue culture infective dose (TCID_50_) was calculated according to Reed and Muench [6]. Vero E6 TMPRSS-2 (provided by Stefan Pöhlmann; Deutsches Primatenzentrum, Göttingen, Germany)—initially described in Hoffmann et al. [^7^]—were cultured in Dulbecco’s modified Eagle’s medium (DMEM) with 10% fetal bovine serum (FBS) and used for virus passaging and isolating infectious virus from clinical samples. The virus used for the neutralization assay was originally isolated from a clinical specimen (NP swab), taken in mid-March 2020 from a 25-year-old male patient in Lower Austria and further passaged twice on Vero E6 TMPRSS-2 cells [7].

### Virus isolation from clinical samples

Vero E6 TMPRSS-2 cells were seeded in 12- or 24-well tissue culture plates to 80% confluency; 400 µl of NP and OP swab supernatant was inoculated on to the monolayer and incubated for 2 h to allow virus entry. After that, the inoculum was removed, the cells washed once with sterile Dulbecco’s phosphate-buffered saline (DPBS) (Lonza, NH, USA) and DMEM added, supplemented with 2% FBS and 1xanti/anti (Life technologies, AT). As positive control, 2000 TCID_50_ of SARS-CoV-2 virus preparation were inoculated at the same time with the samples, but on separate tissue culture plates. After 2 or 3 days, the cells were inspected under an inverted light microscope for any signs of cytopathic effects (CPE). The tissue culture plates were transferred to -80 °C for an hour and subsequently defrosted at room temperature. A maximum of 50% of the first passage was transferred onto a new cell monolayer after sterile filtration of the sample (0.45 µM PES, Minisart, Millipore) to remove cellular debris. After incubation for 2 h, the cleared supernatant of passage 1 was removed, cells were washed with 1xDPBS and fresh medium was added. These steps were repeated for up to four passages.

### ELISA

Sera were tested with COVID-19 ELISA IgG test kit (Vircell, Spain) and ID Screen ® SARS-CoV-2-N IgG Indirect ELISA (IDvet, France). All samples were thermally inactivated (water bath, 56 °C, 60 min) and tested in technical duplicates. Optical density (OD) was measured by the ELISA reader ELx808 (BioTek Instruments, Inc., VT, USA) using the included software “Gen5” version 3.08 (BioTek Instruments). Tests were performed and analyzed following the producer’s instructions: for the COVID-19 ELISA IgG test kit, an antibody index > 6 was considered positive. In the ID Screen ® SARS-CoV-2-N IgG Indirect ELISA samples presenting a sample to positive ratio (S/P%) > 70 were considered positive.

### Serum neutralization test (SNT)

The neutralization assay was set up in flat-bottom 96-well tissue culture plates. Human sera were heat-treated for 30 min at 56 °C and diluted 1–10 in serum-free DMEM medium as a starting point for the assay. Twofold serially diluted sera were incubated with an equal volume of 50 μl SARS-CoV-2 at a minimum of 2000 tissue culture infectious dose 50% (TCID_50)_/ml) for 90 min at 37 °C. Next, 25,000 Vero 76 clone E6 cells were added to the serum/virus mixture in each well in a volume of 100 µl in EMEM, supplemented with 10% FBS and incubated for 4 days at 37 °C, 5% CO_2_ in a humidified incubator. The CPE in every well was scored under an inverted optical microscope and the reciprocal of the highest serum dilution that protected more than 50% of cells from CPE was taken as the neutralizing titer.

## Results

Twenty-three of the forty invited HCW agreed to participate in the study and attended the outpatient clinic of the Kaiser Franz Josef Hospital.

Among these, eight yielded negative NP and OP RT-PCR results on samples gained during their first study visit at the outpatient clinic. Among the 15 remaining study participants, four (27%) were males and eleven (73%) females. Their median age was 41 years (mean 43 years; range 22–59 years). Four of the 15 HCWs (27%) had associated co-morbidities, including two persons with treated arterial hypertension, two with substituted hypothyroidism and one with well-controlled bronchial asthma. None of the study participants had been hospitalized during their SARS-CoV-2 infection; two of 15 (13%) have had an asymptomatic and 13 of 15 (87%) a mild course of the disease. The median duration of RT-PCR test positivity was 23 days (range 5–51 days), the median duration between symptom onset and first study visit was 37 days (range 19–58 days). Evaluation of the test subject’s medical records revealed the occurrence of alternating positive and negative PCR results from previously taken swabs for 6 (40%) of these 15 HCWs. Among the 13 symptomatic participants, the most common symptoms were fever (11; 85%), cough (5; 33%), dysosmia (9; 60%) and dysgeusia (10; 67%). The basic demographic, clinical and diagnostic characteristics of the study participants are summarized in Table 1.

**Table 1:** Overview

Characteristics	All HCWs (n = 15)
Median age—years (range)	41 (22–59)
Female sex—no. (%)	11 (73%)
Co-morbidity—no. (%)	4 (27%)
Symptomatic course of disease—no. (%)	13 (87%)
Median duration of symptoms—days (range)	10 (0–38)
Median duration of positive RT-PCR—days (range)	23 (5–51)
Positive IgG-ELISA serology—no. (%)	15 (100%)
Positive serum neutralization assay—no. (%)	14 (93%)

This table shows the basic demographic, clinical and diagnostic characteristics of the 15 study participants who visited the outpatient clinic between April 30 and June 11, 2020

Abbreviations: no., number; RT-PCR, reverse transcription polymerase chain reaction; IgG, immunoglobulin G; ELISA, enzyme-linked immunosorbent assay

Of 58 respiratory specimen swabs, 24 yielded a positive result for viral RNA in RT-PCR (Table 2); 14 positive samples were detected in NP and 10 in OP swabs. Five persons had detectable viral RNA in OP and NP samples taken at the same time. Among all 58 respiratory specimen swabs, median threshold cycle was 37.2 (mean 37.3; range 30.8–41.6) for NP swabs and 37.6 (mean 37.6; range 34.5–40) for OP swabs.

**Table 2:** Detailed characteristics of all study visits

Baseline characteristics				First study visit						Second study visit						Third study visit					
ID no	Sex	Age	Duration of PCR positivity	Days after symptom onset	IgG ELISA titer Vircell ®	IgG ELISA titer IDVET ®	SNT titer	NP PCR (Ct)	OP PCR (Ct)	Days after symptom onset	IgG ELISA titer Vircell ®	IgG ELISA titer IDVET ®	SNT titer	NP PCR (Ct)	OP PCR (Ct)	Days after symptom onset	IgG ELISA titer Vircell ®	IgG ELISA titer IDVET ®	SNT titer	NP PCR (Ct)	OP PCR (Ct)
1	f	50	20	21	52	292	30	36.5	no Ct												
3	f	39	25	33	28	222	< 10	37.8	40	40	29	217	15	no Ct	no Ct						
4	f	52	45	45	54	317	40	36.6	no Ct	52	64	344	120	no Ct	38	59	54	316	120	no Ct	no Ct
7	m	55	37	37	53	329	30	39	no Ct	44	*	*	*	no Ct	no Ct						
8	f	41	22	31	29	211	30	37	no Ct	38	19	199	40	no Ct	no Ct						
9	f	39	51	44	29	245	< 10	37	no Ct	58	21	217	20	37.4	no Ct	72	26	186	20	no Ct	no Ct
11	m	41	17	–	11	118	< 10	41.3	no Ct	–	11	121	< 10	no Ct	no Ct						
12	f	22	36	38	19	154	25	no Ct	37.3	45	18	155	15	no Ct	no Ct						
13	m	37	14	–	9	72	0	no Ct	41.7	–	10	68	0	no Ct	no Ct						
15	m	59	5	58	23	237	160	39.8	34.5	65	26	235	160	no Ct	no Ct						
16	f	32	20	20	16	218	120	34	no Ct												
17	f	36	23	23	46	315	40	39.7	no Ct	30	53	287	80	no Ct	no Ct						
18	f	32	38	38	21	139	< 10	41.6	35.3	45	21	129	10	no Ct	39.1						
19	f	49	14	19	39	163	20	30.8	35.1	26	42	222	40	33.4	34.8						
23	f	56	46	49	> 60	288	240	no Ct	39.9												

This table demonstrates all study visits of the participant HCWs between April 30 and June 29, 2020, including follow-up appointments

Abbreviations: ID no., identification number; IgG ELISA, immunoglobulin G enzyme-linked immunsorbent assay NP, nasopharyngeal; OP, oropharyngeal; PCR, polymerase chain reaction; Ct, threshold cycle; SNT, serum neutralization test

^*^ No blood sample obtained

- Asymptomatic course of disease

13 of the 15 participants RT-PCR positive at the first study visit, appeared at a follow-up after 7 days (second study visit); of these, 4 persons again tested positive by RT-PCR of respiratory swabs. Among those four HCWs, two agreed on a third study visit—one after 7, the other one after 14 days; no viral nucleic acid was detected in either.

Infectious virus was not detected in any culture of the 58 respiratory specimens over the entire period of the study. The presence of IgG antibodies was observed in all 15 RT-PCR positive HCWs, as seen in Table 2. Neutralizing antibodies were detected in 7 of 15 HCW at the first visit and in 14 of 15 HCWs at their second study visit. A Spearman-Rho correlation analysis was performed showing a significant correlation between the level of IgG in ELISA and level of neutralizing antibodies. The correlation was shown for both the IgG Vircell ELISA (*p* = 0.003) and the IgG IDvet ELISA (*p* < 0.001). Furthermore, a *t* test analysis showed a significant correlation between the level of IgG antibodies in both test kits and the SNT result (*p* < 0.001); in samples with negative SNT results, the median level of IgG antibodies was 18.5 (Vircell IgG ELISA; range 9–29, SD 8.1) and 150 (IDvet IgG ELISA; range 68–245, SD 61.3), while samples with positive SNT results showed median levels of 35.8 (range 10–64, 17.3) and 231 (range 68–344, SD 74.7).

## Discussion

In this study, we demonstrate that despite RT-PCR positivity, no infectious virus could be isolated from respiratory swab samples taken from convalescent HCWs.

Our findings agree with the literature [3, 4] indicating that even after a mild course of COVID-19 nucleic acid of SARS-CoV-2 can still be detected by RT-PCR for several weeks after symptoms have already subsided. In our study, the longest duration of SARS-CoV-2 nucleic acid shedding was 58 days after illness onset and 51 days after the patients’ first positive RT-PCR result.

We observed a higher sensitivity for nasopharyngeal swabs than for oropharyngeal swabs. This contrasts with a study by Woelfel et al. (2020) [8] who described no discernible differences concerning detection rates when comparing NP and OP swabs in nine patients hospitalized with COVID-19. However, their swabs were only taken during days 1–5 of symptoms. Our results are in accordance with the findings of Yang et al. (2020) which also indicated that SARS-CoV-2 is less frequently detected in OP than in NP swabs: the differences in detection rate increase with the time passed since symptom onset, particularly from day 8 onwards [9].

Viral RNA shedding of SARS-CoV-2 does not equate with infectivity unless there is proof that the virus can be isolated and cultured from the particular samples [2]. In our study, no viable virus was detected in either NP or OP swabs. This is in line with findings from Woelfel et al. (2020) [8] who found that virus isolation probability was low when the SARS-CoV-2 RNA load was below 5.4 log^10^ copies/swab, which corresponds with a Ct-value of about 29.5 in their RT-PCR system [10].

Our findings are also consistent with the quantitative data gained in a Chinese study, showing a high viral load of SARS-CoV-2 in the upper respiratory tract around the time of symptom onset, followed by a gradual decrease to low levels in the second week [11]. In a Canadian study, SARS-CoV-2 confirmed positive throat samples were tested for their ability to infect Vero cell lines, like we did, to determine infectivity [12]. Again, it was shown that infectivity was only observed in throat samples from persons with symptoms lasting fewer than 8 days.

A French study group obtained 183 samples (174 nasopharyngeal swabs and 9 sputum samples) from 155 patients and inoculated them in cell cultures to correlate viral load to cultivable viruses. No positive culture result was obtained from samples with Ct value of > 34. Based on these data, La Scola et al. deduced that patients with Ct values > 34 do not excrete infectious viral particles and may thus be discharged [13].

In our study, only one of the 15 volunteers yielded positive PCR results with Ct values below 34. Test subject no. 19 showed a threshold cycle of 30.8 from a NP swab taken on day 19 after symptom onset (neutralization assay titer at this time: 1:20) and a threshold cycle of 33.4 with a NP swab gained 26 days after symptom onset (neutralization assay titer at this time 1:40). However, infectious virus was not isolated from any of these swabs.

Based on results gained from 19 Canadian cases, Bullard et al. concluded that respiratory samples from COVID-19 patients at ≥ 8 days post symptom onset and a SARS-CoV-2 E-gene RT-PCR value > 24 (!) may predict lack of infectivity; of 90 RT-PCR SARS-CoV-2-positive samples incubated on Vero cells, there was no growth in samples with a Ct > 24 or symptom onset to viral culture test time > 8 days. They state, that for every unit increase in Ct, the odds ratio for infectivity decreases by 32% [12]. Based on the studies mentioned above, recent recommendations of the federal public health authority in Germany (Robert Koch Institute) for the cessation of isolation of HCWs with severe COVID-19, i.e. requiring oxygen, include a Ct value of > 30 [14].

In our study, we could not detect infectious virus in any of 29 nasopharyngeal and 29 oropharyngeal swabs from 15 long-time carriers. Currently, it is not clear what role neutralizing antibodies (NAbs) play during a SARS-CoV-2 infection [15]. NAb titers in the early stages of the infection are inversely correlated with subsequent viral loads, measured as RNA copies in sputum and throat swabs, but are directly correlated with a more severe subsequent disease [8]. People with mildly symptomatic infections not requiring hospitalization generally have far weaker antibody responses than patients who develop the most severe disease, also in the oldest ones [16, 17]. Wu et al. tested 175 patients who had recovered from mildly symptomatic COVID-19; in 10 cases (5.7%) NAbs were undetectable [18]. This percentage correlates very well with our finding, where 1/13 (7.7%) participants lacked any detectable NAbs. Undetectable NAbs must not be misunderstood as evidence for lack of protection from SARS-CoV-2 infection and/or severe disease. Gallais et al. recently stated, that some contacts of patients with COVID-19, who failed to seroconvert, already show evidence of T-cell response to SARS-CoV-2, suggesting the development of non-humoral immunity or prior T-cell immunity due to a previous infection with other coronaviruses [19].

The limitations of our study include the small sample size, the lack of persons with a severe clinical course as well as the short follow-up.

According to PCR results not gained in our study, but documented in our study participants’ medical records, 6/15 initially positive HCWs converted to negative and later reverted again to positive. This pattern may be expected in the course of an infection, especially for patients with low SARS-CoV-2 RNA loads near the limit of detection. Among their 10,643 cases, Green et al. identified 49 initially positive patients who converted to negative and later reverted to positive. Such cases likely represent the persistence of viral RNA at low levels and demonstrate that previous repeatedly negative results cannot completely rule out a subsequent positive test [20]. Green et al. conclude that for patients with a positive SARS-CoV-2 molecular assay result, repeating the test before at least 15 days after the first test is unlikely to yield a negative result. According to ECDC guidance, for asymptomatic SARS-CoV-2-infected persons, the tests to document virus clearance should be taken a minimum of 14 days after the initial positive test [21].

Virological findings predicting lack of infectivity already after day 8 [8, 12] are supported by epidemiological data in household contact studies, demonstrating that the serial interval was 4–5 days [22, 23], with no transmission after 6 days from symptom onset [23].

Although there is no specific evidence for COVID-19, immunocompromised patients may shed SARS-CoV-2 viral RNA for prolonged periods, similar to other respiratory viruses. However, viral RNA shedding of SARS-CoV-2 does not equate with infectivity, unless there is proof that the virus can be isolated and cultured from the particular samples [2]. Our finding of substantially higher Cts in virus isolation negative, convalescent HCWs as compared to virus culture-positive patients further supports the hypothesis that repeat positivity in convalescent persons represents the detection of non-viable virus material, rather than an active infection.

For several other viral infections, like influenza or measles, a long period of detection of nucleic acid in recovered patients by RT-PCR with simultaneous lack of infectivity is well documented [24, 25]. However, no prolonged isolation of persons recovered from such viral infections is demanded by public health authorities. Based on our findings of the absence of culturable virus in respiratory swabs from convalescent SARS-CoV-2 RNA shedders, we recommend revising present guidelines; we propose de-isolation of HCWs clinically recovered from COVID-19, despite positive RT-PCR results, from swabs gained after at least 8 days of isolation. If the threshold cycle of the RT-PCR of a naso- or oropharyngeal swab is over 30, the person can be considered non-infectious and isolation can be repealed. Positive results obtained from genes targeted with Ct-values over 30 are most likely due to non-viable/noninfectious particles that are still detected by RT-PCR. In case of a threshold cycle, lower than or equal to 30, a serum neutralization test (SNT) should be performed on the convalescent HCW. If the latter is positive, non-infectiousness could be assumed. We acknowledge that SNT is not available in all laboratories and is time-consuming and expensive. Our data suggest that there is a correlation between levels of IgG antibodies and the probability of detecting neutralizing antibodies. However, no ELISA cut-off level could be determined. Further studies will be needed to show the suitability of IgG antibody levels for the determination of infectiousness.

## References

[1] Kreidl P, Schmid D, Maritschnik S, et al. Emergence of Coronavirus disease 2019 (COVID-19) in Austria. Wien Klin Wochenschr. 2020; in press. 10.1007/s00508-020-01723-9 PMC7439636 32816114

[2] European Centre for Disease Prevention and Control. Guidance for discharge and ending isolation in the context of widespread community transmission of COVID-19—first update. ECDC 2020. https://www.ecdc.europa.eu/sites/default/files/documents/covid-19-guidance-discharge-and-ending-isolation-first%2520update.pdf. Accessed 19 Mai 2020.

[3] Li N, Wang X, Lv T (2020). Prolonged SARS-CoV-2 RNA shedding: not a rare phenomenon. J Med Virol..

[4] Zhou F, Yu T, Du R (2020). Clinical course and risk factors for mortality for adult inpatients with COVID-19 in Wuhan, China: a retrospective cohort study. Lancet.

[5] Toussaint JF, Sailleau C, Breard E (2007). Bluetongue virus detection by two real-time RT-qPCRs targeting two different genomic segments. J Virol Methods..

[6] Reed LJ, Muench H (1938). A simple method of estimating fifty percent endpoints. Am J Epidemiol..

[7] Hoffmann M, Kleine-Weber H, Schroeder S (2020). SARS-CoV-2 cell entry depends on ACE2 and TMPRSS2 and is blocked by a clinically proven protease inhibitor. Cell.

[8] Wölfel R, Corman VM, Guggemos W (2020). Virological assessment of hospitalized patients with COVID-2019. Nature.

[9] Yang Y, Yang M, Shen C, et al. Evaluating the accuracy of different respiratory specimens in the laboratory diagnosis and monitoring the viral shedding of 2019-nCoV infections. medRxiv. 2020; https://doi.org/10.1101/2020.02.11.20021493.

[10] Jones TC, Mühlemann B, Veith T, et al. An analysis of SARS-CoV-2 viral load by patient age. medRxiv. 2020; 10.1101/2020.06.08.20125484.

[11] Zou L, Ruan F, Huang M (2020). SARS-CoV-2 viral load in upper respiratory specimens of infected patients. N Engl J Med..

[12] Bullard J, Dust K, Funk D (2020). Predicting infectious SARS-CoV-2 from diagnostic samples. Clin Infect Dis..

[13] La Scola B, Le Bideau M, Andreani J (2020). Viral RNA load as determined by cell culture as a management tool for discharge of SARS-CoV-2 patients from infectious disease wards. Eur J Clin Microbiol Infect Dis..

[14] Robert Koch Institute (RKI). COVID-19: Entlassungskriterien aus der Isolierung. Orientierungshilfe für Ärztinnen und Ärzte (2.7.2020) [COVID-19: Criteria for cessation of isolation. Guidance for physicians. (2 July 2020)]. Berlin: RKI 2020. https://www.rki.de/DE/Content/InfAZ/N/Neuartiges_Coronavirus/Entlassmanagement.html. Accessed 2 Juli 2 2020.

[15] Moore JP, Klasse PJ (2020). SARS-CoV-2 vaccines: ‘Warp speed” needs mind melds not warped minds. J Virol..

[16] Long Q-X, Tang X-J, Shi Q-L (2020). Clinical and immunological assessment of asymptomatic SARS-CoV-2 infections. Nat Med..

[17] Robbiani DR, Gaebler C, Muecksch F (2020). Convergent antibody responses to SARS-CoV-2 infection in convalescent individuals. BioRxiv..

[18] Wu F, Wand A, Liu M, et al. Neutralizing antibody responses to SARS-CoV-2 in a COVID-19 recovered patient cohort and their implications. medRxiv. 2020; 10.1101/2020.03.30.20047365.

[19] Gallais F, Velay A, Wendling MJ, et al. Intrafamilial exposure to SARS-CoV-2 induces cellular immune response without seroconversion. medRxiv 2020; 10.1101/2020.06.21.20132449. 10.3201/eid2701.203611 PMC7774579 33261718

[20] Green DA, Jason Z, Westblade LF (2020). Clinical performance of SARS-CoV-2 molecular testing. J Clin Microbiol..

[21] European Centre for Disease Prevention and Control (ECDC). Technical report. novel coronavirus (SARS-CoV-2): Discharge criteria for confirmed COVID-19 cases—When is it safe to discharge COVID-19 cases from the hospital or end home isolation? Stockholm: ECDC 2020. https://www.ecdc.europa.eu/sites/default/files/documents/COVID-19-Discharge-criteria.pdf. Accessed 29 June 2020.

[22] Cheng H-Y, Jian S-W, Liu D-P (2020). Contact tracing assessment of COVID-19 transmission dynamics in Taiwan and risk at different exposure periods before and after symptom onset. JAMA Intern Med..

[23] Nishiura H, Linton NM, Akhmetzhanov AR (2020). Serial interval of novel coronavirus (COVID-19) infections. Int J Infect Dis..

[24] Zitterkopf NL, Leekha S, Espy MJ (2006). Relevance of influenza a virus detection by PCR, shell vial assay, and tube cell culture to rapid reporting procedures. J Clin Microbiol..

[25] Woo GK, Wong AH, Lee WY (2010). Comparison of laboratory diagnostic methods for measles infection and identification of measles virus genotypes in Hong Kong. J Med Virol..

